# Symptomatic anterior cruciate ligament tears treated with percutaneous injection of autologous bone marrow concentrate and platelet products: a non-controlled registry study

**DOI:** 10.1186/s12967-018-1623-3

**Published:** 2018-09-03

**Authors:** Christopher Centeno, Jason Markle, Ehren Dodson, Ian Stemper, Christopher Williams, Matthew Hyzy, Thomas Ichim, Michael Freeman

**Affiliations:** 1 Centeno-Schultz Clinic, 403 Summit Blvd Suite 201, Broomfield, CO 80021 USA; 2 Regenexx, LLC, Des Moines, IA 50321 USA; 3 Immune Advisors, LLC, San Diego, CA USA; 4 CAPHRI School of Public Health and Primary Care, Maastricht University, Maastricht, The Netherlands

**Keywords:** Anterior cruciate ligament, ACL, Bone marrow concentrate, BMC, Mesenchymal stem cells, MSC, Percutaneous injections, Regenerative medicine, Knee instability, Knee injury

## Abstract

**Background:**

Bone marrow concentrate (BMC) has shown promise in the treatment of several orthopedic conditions. This registry study investigated the use of autologous BMC and platelet products for percutaneous anterior cruciate ligament (ACL) treatment.

**Methods:**

Twenty-nine patients presenting to a single outpatient interventional musculoskeletal and pain practice with symptomatic grade 1, 2, or 3 ACL tears with less than 1 cm retraction were enrolled. Patients were treated with a percutaneous ACL injection of autologous BMC and platelet products using fluoroscopic guidance. Pre- and post-treatment magnetic resonance imaging analysis was completed for 23 patients using ImageJ software for an objective quantitative analysis of pixel density as a proxy for ACL integrity. Subjective clinical outcome measures collected pre-treatment and at 1, 3, 6, 12, 18, 24, and 36 months post-treatment include the Numerical Pain Scale (NPS), the Lower Extremity Functional Scale (LEFS), the International Knee Documentation Committee (IKDC) form, and a modified version of the Single Assessment Numeric Evaluation.

**Results:**

Seventy-seven percent of patients treated with BMC injections into the ACL showed significant improvement (p < 0.01) in objective measures of ACL integrity at an average of 8.8 months (median 4.7 months). The mean of last patient-reported improvement was 72% (SD = 35) at an average of 23 (SD = 10) months post-treatment. Mean scores were found to be significantly different (p < 0.05) for the NPS at 6, 18, and 24 months, and LEFS and IKDC at all time points (i.e. 1, 3, 6, 12, 18, 24, and 36 months) relative to baseline.

**Conclusion:**

In symptomatic patients with grade 1, 2, or even grade 3 tears with minimal retraction, ACL treatment with percutaneous injection of BMC and platelet products shows promise as a non-surgical alternative. However, a larger randomized controlled trial is warranted to confirm these findings.

*Trial registration* NCT03011398. A Clinical Registry of Orthobiologics Procedures. https://clinicaltrials.gov/ct2/show/NCT03011398?term=orthobiologics&rank=1. Registered 29 December 2016. Enrollment 1 December 2011-retrospectively registered

## Background

The anterior cruciate ligament (ACL) is an important stabilizer of the knee, limiting anterior translation and rotation of the tibia [1]. Stability of the ACL is created by two major bundles of the ligament, the anteromedial bundle and posterolateral bundle. The ACL is the most commonly injured ligament in the knee, with 200,000 ACL reconstructions performed in the United States each year at an estimated cost of $2–3 billion [2, 3]. Rupture of the ACL is characterized by joint instability, resulting in decreased activity, poor knee biomechanics, and decreased quality of life [4, 5]. Partial ACL tears can be addressed without surgical reconstruction with good short- and medium-term functional results, particularly if sports participation is restricted [6]. Complete tears, on the other hand, result in long-term instability in 15–66% of patients and are associated with a 15–86% risk of a subsequent meniscal tear [7].

Surgical reconstruction of the ACL is commonly used for treating ACL tears when there is persistent knee instability and functional limitation, or for athletes who will be returning to lateral, pivoting sports (e.g. soccer, basketball, etc.) [1, 6]. Reconstruction typically involves removing a portion of the patient’s patella or hamstring tendon (autograft) or using a donor cadaver tendon (allograft) as a replacement for the ACL. The remaining native ACL is removed and graft anchoring sites are created by drilling into and through the lateral femoral condyle and tibial plateau. The procedure is not universally successful with 15–25% of patients noting persisting pain and instability post-operatively [1].

There are disadvantages to ACL reconstruction surgery even when successful. Typically, the anchoring sites position the ACL graft at a more vertical angle than the native ligament and thus can fail to prevent excessive anterior motion of the tibia relative to the femur, while also increasing compression forces on the joint surfaces. Such post-surgical biomechanical alterations of the knee likely contribute to accelerating development of degenerative osteoarthritis (OA) in the joint [8]. Long-term results of ACL reconstruction among teens aged 10–16 years shows 67% of patients had developed OA by age 30 [9]. Other issues include strength loss in the muscle serving as the autograft donor site [10]. Post-surgical strength deficits combined with the single bundle configuration of the ligament graft can allow increased tibial rotation, which, in addition to impaired neuromuscular control, can lead to increased risk of re-injury [9]. In addition, the removal of the native ACL during reconstruction can lead to proprioception impairments; proprioceptive acuity has been found to strongly correlate with patient satisfaction post-surgery [11, 12].

For these and other reasons, non-surgical alternatives to reconstruction are an attractive therapeutic option for ACL injuries. Our group previously described a promising treatment for ACL injury, in which percutaneously injected bone marrow concentrate (BMC) is used in an attempt to help the injured ligament heal [13]. There is basic science evidence that supports this procedure could accomplish ligament healing. Several studies using animal models and various stem cell types found in bone marrow demonstrate ligament and ligament-bone interface healing [14]. Additionally, mesenchymal stem cells (MSC) have been identified in injured ACL remnants in human patients, suggesting that MSCs are likely involved in natural attempts at ligament healing [15].

This current study expands from our previously published case series on a larger sample of patients with ACL injury treated with autologous BMC and platelet products. The purpose of this larger study is to document the use of this procedure prior to embarking on a randomized controlled trial, further assessing magnetic resonance imaging (MRI) healing using an updated normalization technique, comparing pre- and post-treatment MRIs to uninjured ACL MRIs, and analyzing outcomes between ACL tear grades.

## Methods

### Study design and clinical protocol

The study sample consisted of consecutive patients presenting to a single outpatient interventional musculoskeletal and pain practice between December 2011 and May 2015 for evaluation of complaints of knee instability combined with MRI evidence of an ACL tear. Patients who were diagnosed with a functional disability and significant ligamentous laxity on examination with Lachman testing (in comparison with the uninvolved side) were eligible for enrollment, and tracked over time via a treatment registry.

Inclusion criteria:

- Patients agreeing to enroll in the treatment registry and undergo BMC and platelet products treatment, who displayed a grade 1, 2, or 3 ACL tear on MRI (as defined below). If a high-grade tear, only those with less than 1 cm of ligament retraction were included.
- No limitation on duration of injury.

Exclusion criteria:

- Patients younger than 15 years
- Active neoplasm within the past 5 years
- Anemia
- Grade 3 ACL tear with > 1 cm retraction.

Patients provided verbal and written consent. The International Cellular Medicine Society provided Institutional Review Board (IRB) oversight and approval (OHRP Registration #IRB00002637).

ACL injuries were graded as follows [16]:

- Grade 1 sprain: the ligament is partially torn, with less than half of the ligament substance disrupted
- Grade 2 sprain: the ligament is partially torn, with more than half of the ligament substance disrupted
- Grade 3 sprain: the ligament is completely torn.

### Bone marrow aspiration and concentration

For a more detailed description of the bone marrow aspiration (BMA) and injection procedures used in the present study, please see our prior publication [13]. In brief, 2 weeks before the procedure, patients were asked to refrain from taking non-steroidal anti-inflammatory drugs and corticosteroids due to the potential adverse effects on healing and MSC activity [21].

Bone marrow was harvested from the patient’s posterior superior iliac crest on the same day as the therapeutic step of the procedure. A total of approximately 60–120 cc of whole bone marrow aspirate was removed from 6 to 10 sites under ultrasound or fluoroscopic guidance. Draw volume tended to vary with patient size.

Under sterile conditions, the aspirate was centrifuged to isolate the buffy coat, which was processed manually in class II type A2 biological safety cabinet to produce 2–5 cc of BMC for injection to the treatment site in the knee. Total nucleated cells were counted from the bone marrow aspirate.

### Preparation of platelet products

**Fig. 1:**
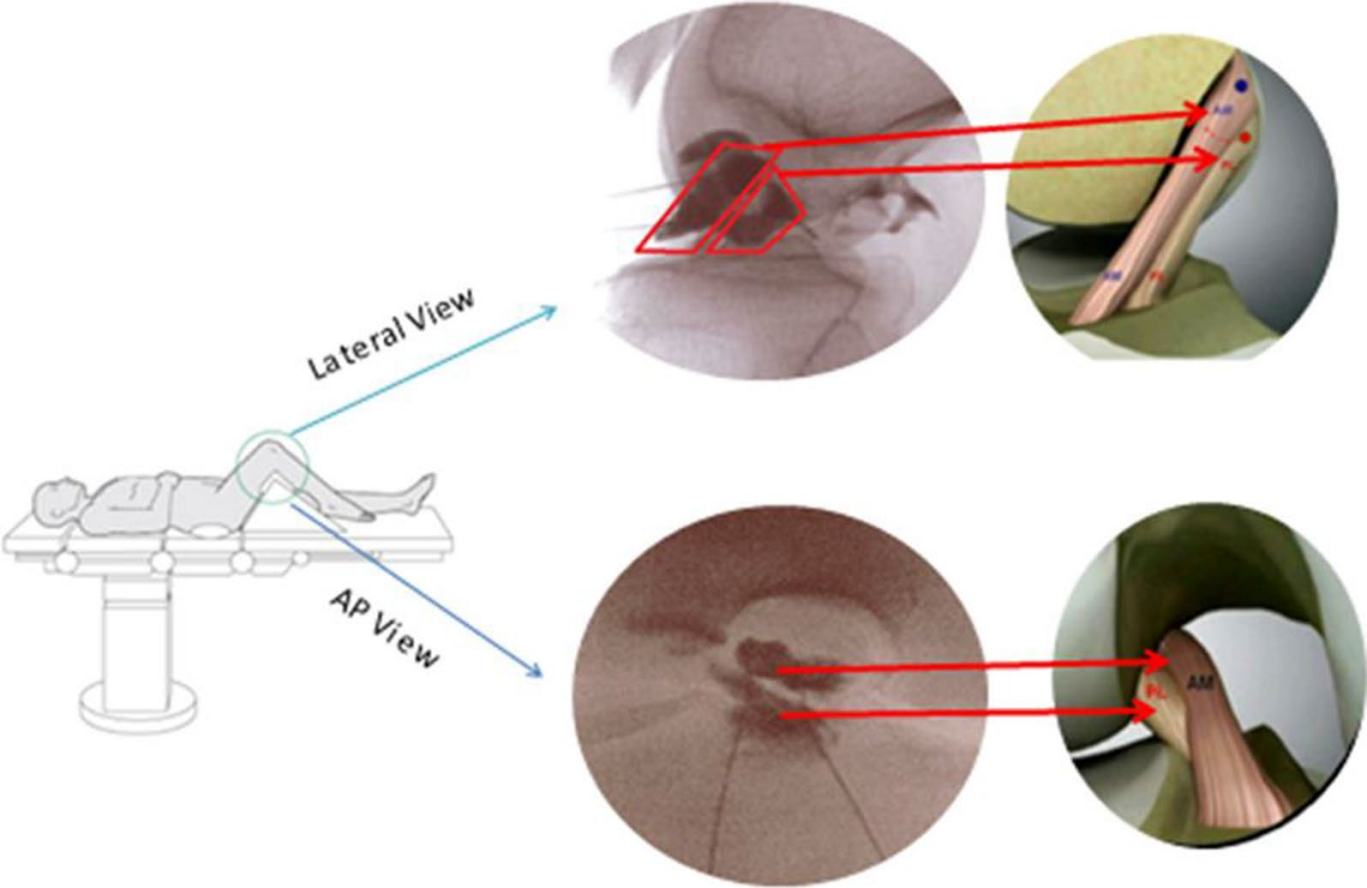
ACL double bundle injection. Patient is placed in supine position on exam table with target knee bent (as shown) to obtain AP and lateral views of the ACL. Using fluoroscopy, two separate 25 gauge 3.5 in. needles are inserted toward the origin and insertion of ACL. Once needles are in correct location, specific bundles are targeted with contrast dye outlining each bundle and placing bone marrow concentrate into each location

Concurrently, 60 cc of venous blood was drawn from the patient using ACD-A collection tubes from which platelet-rich plasma (PRP) was isolated and divided into PRP for injection and PRP for further processing. Platelet lysate (PL) was then prepared from the PRP via freeze-thawing, which initiates lysis of the platelets [17]. Using fluoroscopy to guide needle placement, the solution consisting of 2–3 cc of BMC, PRP and PL was injected directly into the ligament after contrast (Iodexidol, NDC#0407-2223-06) flow was used to indicate appropriate placement. The needle was withdrawn from the ligament approximately 1 cm, and while still in the joint, approximately 2–4 cc of a mixture of 1–1 cc of PRP and PL along with any remaining BMC were injected into the joint. Figure 1 displays how the specific injection technique targets the two bundles of the ACL using fluoroscopy. The addition of PRP and PL was to help augment the healing response with additional growth factors to aid in tissue repair and healing via the proliferation of MSCs contained in BMC [18, 19].

### Injection protocol and timing

The BMC injection described above was preceded by a pro-inflammatory pre-injection of hyper-osmolar dextrose 2–5 days before the procedure. The pre-injection creates a pro-inflammatory and proliferative environment for fibroblasts in the ACL, as the dextrose solution causes a chemical micro-injury in the ligament. The spacing of the injections several days apart allows sufficient time for the cellular phase of healing to commence prior to the BMC injection [20]. Eight patients did not receive the pre-injection, as part of a separate protocol.

After the procedure, patients were instructed to engage in activity as tolerated. Post-treatment bracing was not used. Patients were encouraged to undergo physical therapy, but this was neither controlled nor required.

### Clinical outcome measures

Patients were enrolled in a treatment registry (#IRB00002637) and tracked prospectively using the electronic database system ClinCapture (Clinovo Clinical Data Solutions, Sunnyvale, CA, USA) (http://www.clinovo.com/clincapture). Patient-reported outcome questionnaires included a Numerical Pain Scale (NPS), the Lower Extremity Functional Scale (LEFS) [21] and the International Knee Documentation Committee (IKDC) form [22], which were collected pre-treatment and at 1, 3, 6, 12, 18, 24, and 36 months post-treatment. Patients were contacted via email and phone up to five times at each time point.

The NPS is a segmented version of the visual analog scale ranging from 0 to 10, where “0” means no pain and “10” means the worst possible pain. The LEFS is a 20-item questionnaire that assesses a person’s lower-extremity functional ability to perform everyday tasks, with scores ranging from 0 (extreme disability) to 80 (no functional disability). The IKDC measures knee-specific symptoms, daily function and ability to perform sports activity in patients with knee injuries or conditions, with total scores ranging from 0 (extreme disability) to 100 (no functional disability). Change scores were created for NPS, LEFS and IKDC by taking the difference between patients’ outcome scores at baseline and their scores at each post-treatment time point.

A modified version of the subjective Single Assessment Numeric Evaluation (SANE) rating was also administered [23]. The modified version used in the present investigation was the patient’s response to the question “Compared to your condition prior to the procedure, what percent difference have you seen in your condition?” The modification of the SANE used for our study allowed for the patient to report worsening of condition, as well as improvement, with a range of − 100% indicating maximum subjective worsening to + 100% indicating maximum subjective improvement, and 0% indicating no change. Any negative score was adjusted to 0 to match the commonly reported SANE scale.

### Pre- and post-treatment imaging analysis

Magnetic resonance imaging gray scale measurements were analyzed using ImageJ to assess quantitative changes in signal intensity of the ACL on MRI at the post-treatment time point compared to pre-treatment, as a proxy for ligament integrity. ImageJ (version 1.51) is a publicly available Java image processing and analysis program, developed at the National Institutes of Health [24]. The measurements included mean gray value, modal gray value, median gray value, skewness, and raw integrated density. The mean gray value is the sum of the gray values of all the pixels divided by the number of pixels. The modal gray value is the most frequently occurring gray value; the median gray value is the middle value of all pixels. The raw integrated density is the sum of the values in the selection. For all metrics, a lower value was an indication of a darker or denser image, which is considered typical of a normal (uninjured) ACL. Validation of the imaging protocol used for the present investigation was described our previously published pilot study, with excellent intra- and interrater correlations (0.9 to 1.0) [13].

**Fig. 2:**
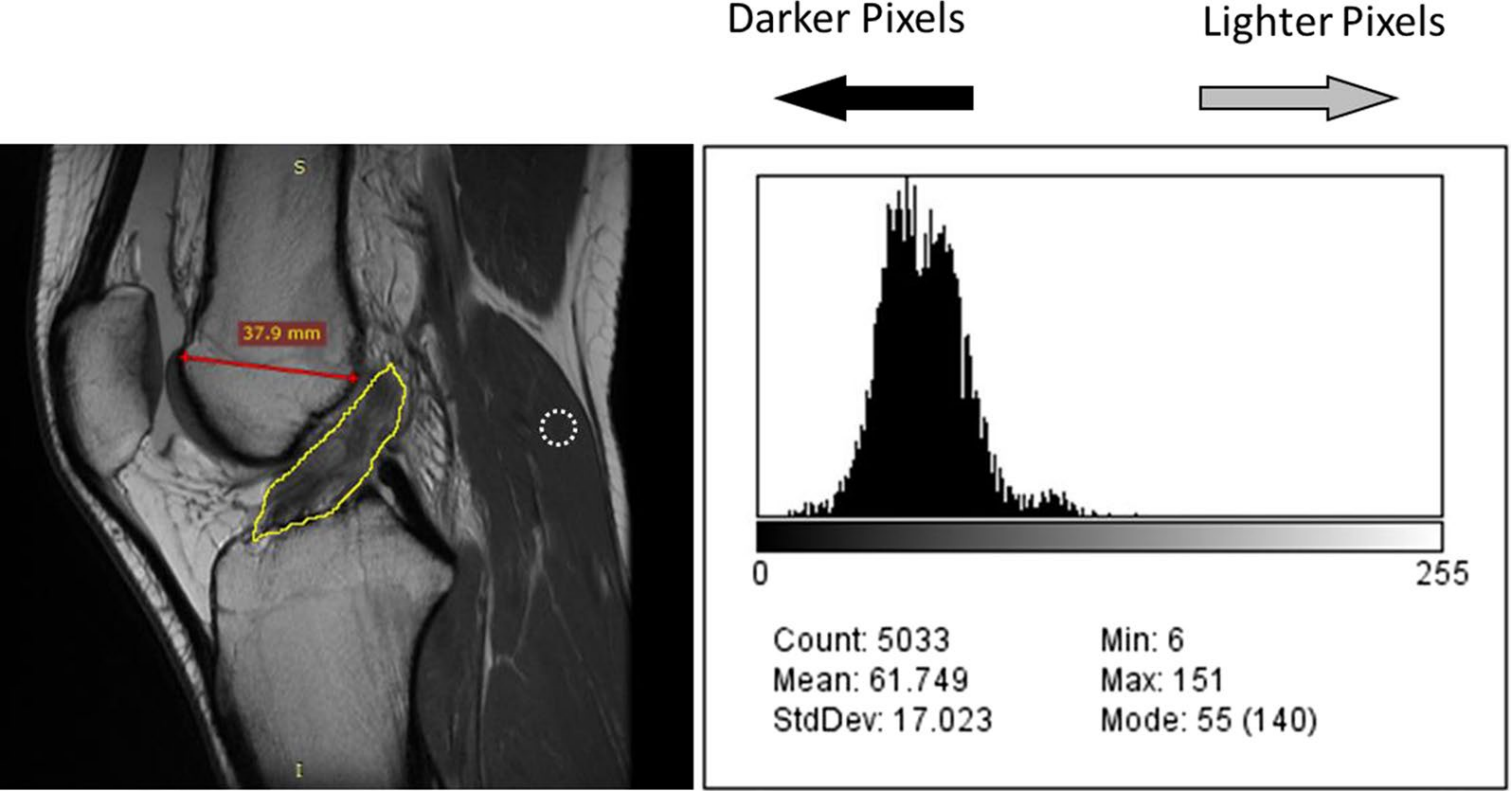
Example of MRI measurements for ImageJ analysis. Outline of the region of interest (ROI) around the ACL in a sagittal MRI view shown here. The resultant histogram to the right shows the frequency of each pixel in the ROI. The x-axis is ordered from darker pixels to the left and lighter to the right. The white dotted circle outline represents a typical outline of the gastrocnemius ROI for normalization. The red line represents the A–P femoral width measurement

Pre- and post-treatment MRIs were collected and analyzed for 23 patients. The sagittal MRI image allowed for the greatest visualization of the cross-sectional area of the ACL and was therefore utilized to evaluate the linear integrity of the ligament fibers. For each patient, the closest matching pre- and post-treatment T − 1 MRI slices were selected from the same sequence and compared side-by-side. In an attempt to ensure accurate MRI slice comparison, anterior–posterior (A–P) femoral head width was measured with the goal of achieving < 1 mm difference between pre- and post-treatment MRI images. The image window function on RadiAnt DICOM viewer was used to match the brightness between images. Within ImageJ, a region of interest (ROI) was created by outlining the ACL on each pre- and post-treatment MRI (see Fig. 2). This ROI was analyzed between the pre- and post-treatment MRIs across the gray scale measurements described previously.

Twelve additional patients with no history of ACL injury and uninjured ACL on MRI were analyzed using the same technique described above. This group’s MRI measurements were used to determine a proxy for uninjured ACL gray value measurements. The hypothesis was that pre-treatment ACL gray value metrics would be significantly higher than uninjured ACLs, while post-treatment ACLs would be more similar to uninjured ACLs.

Inherent differences exist between MRI sessions including different imaging settings or different MRI machines’ Tesla magnet strength. In an attempt to reduce the signal alteration present between imaging sessions, each MRI was normalized to a small ROI on its respective gastrocnemius origin (Fig. 2). This area of the muscle was chosen because it demonstrated consistently very low signal (low mean gray values) across all MRIs. This was done to confirm gray value metric differences between pre- and post-treatment MRIs are indicative of healing and not a result of imaging session variances.

### Adverse events

Complications and adverse events were collected at each follow-up time point and reviewed by the treating physician.

### Statistical analysis

Descriptive characteristics were given as averages with standard deviations (SD) for continuous variables and as numbers with percentages for categorical variables. Linear mixed-effects models were used to test for differences in outcome scores over time for the modified SANE, NPS, LEFS and IKDC. These models were chosen for their robust handling of missing data. If significant differences were present, post hoc Tukey was performed to determine which time points differed. Linear mixed-effects models were also created to assess differences in outcomes between tear grade groups. Change scores were calculated as the difference between baseline and post-treatment scores for LEFS and IKDC. Wilcoxon signed-rank tests were performed to assess differences in gray value measurements between pre- and post-treatment MRIs. A single factor analysis of variance (ANOVA) was performed to assess differences between pre-treatment, post-treatment and uninjured ACL mean gray values, both before and after normalization to the gastrocnemius. An ANOVA was performed to test for differences between pre-treatment, post-treatment, and uninjured MRI gastrocnemius mean gray values. Pearson correlations were used to examine the relationship between differences in mean gray values and LEFS and IKDC change scores. All statistical analyses were performed using R, version 3.3.3, along with the user interface RStudio, version 1.0.136.

## Results

A total of 29 patients (17 males and 12 females) who underwent treatment with autologous BMC and platelet products for ACL injury and entered the patient registry were included in the case series. Twenty-three patients received both pre- and post-treatment MRIs. The patient ages ranged from 15 to 65 (average = 35, SD = 13). BMI ranged from 18.8 to 33.6, with an average of 24.5 (SD = 3.8), see Table 1. The average time between treatment and post-treatment MRI follow-up was 8.7 months (SD = 9.8, range 2.6–42.3 months). Six, 13, and 10 patients were diagnosed with grade 1, 2, and 3 tears, respectively. The average total nucleated cell count (TNCC) from the BMC was 690 million (SD = 328). The average volume of the injectate used for treatment was 3.7 mL (SD = 1.7). Two patients had acute ACL tears, 17 had subacute, and 8 had chronic tears. Two patients were excluded from chronological staging given that there was no definable onset. The shortest time between injury and treatment was 1.7 weeks, and the longest time was 350 weeks. The mean time from injury to treatment was 22.6 weeks (excluding 2 outliers that were beyond 2 years post-injury). The mean time to treatment in those with subacute injuries was 13.7 weeks. Eight patients did not receive the pre-injection, though separate analyses showed these patients did not differ from the remaining 21 in any outcome nor ImageJ metrics and therefore these patients were included in the larger sample.

**Table 1:** Demographic information

Variable	N	Average	SD	Min	Max
Age	29	34.7	13	15	65
BMI	28	24.5	3.8	18.8	33.6
TNCC	29	690 × 10^6^	328 × 10^6^	239 × 10^6^	1684 × 10^6^
Males (%)	17 (59%)				
Females (%)	12 (41%)				
Tear grade					
1	6 (21%)				
2	13 (45%)				
3	10 (34%)				

*N* number of patients, *SD* standard deviation, *BMI* body mass index, *TNCC* total nucleated cell count

### Modified SANE

**Fig. 3:**
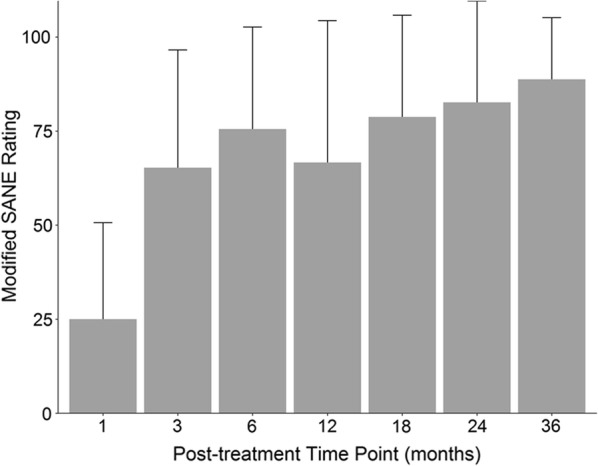
Average self-reported modified SANE ratings at all post-treatment time points with standard deviation bars. Number of patients reporting at each time point: 1 month (N = 14); 3 month (N = 19); 6 month (N = 19); 12 month (N = 21); 18 month (N = 16); 24 month (N = 17); 36 month (N = 8)

The average last reported modified SANE rating was 72% (SD = 35) at an average of 23 months post-treatment (SD = 10). Average post-treatment modified SANE scores ranged from 25% at 1-month post-treatment to 89% at 36 months post-treatment (Fig. 3, with most patients providing data at multiple time points). A linear mixed-effects model showed scores were significantly different across time (p < 0.0001). Post-hoc Tukey showed all time points beyond 1-month post-treatment were significantly higher than 1-month scores. A total of 114 post-treatment modified SANE scores were collected (representing 29 patients), with 89% of scores indicative of improvement (> 0%), and 75% of scores ≥ 50% improvement.

### NPS

Average baseline NPS was 2.5 (SD = 1.8). The last reported post-treatment NPS average was 0.6 (SD = 1.4) at an average of 23 months (SD = 10). A linear mixed-effects model showed scores decreased significantly over time (p < 0.005). Post-hoc Tukey showed mean scores for NPS were significantly lower at the 6, 18, and 24-month time points compared to baseline (p < 0.05).

### LEFS

**Fig. 4:**
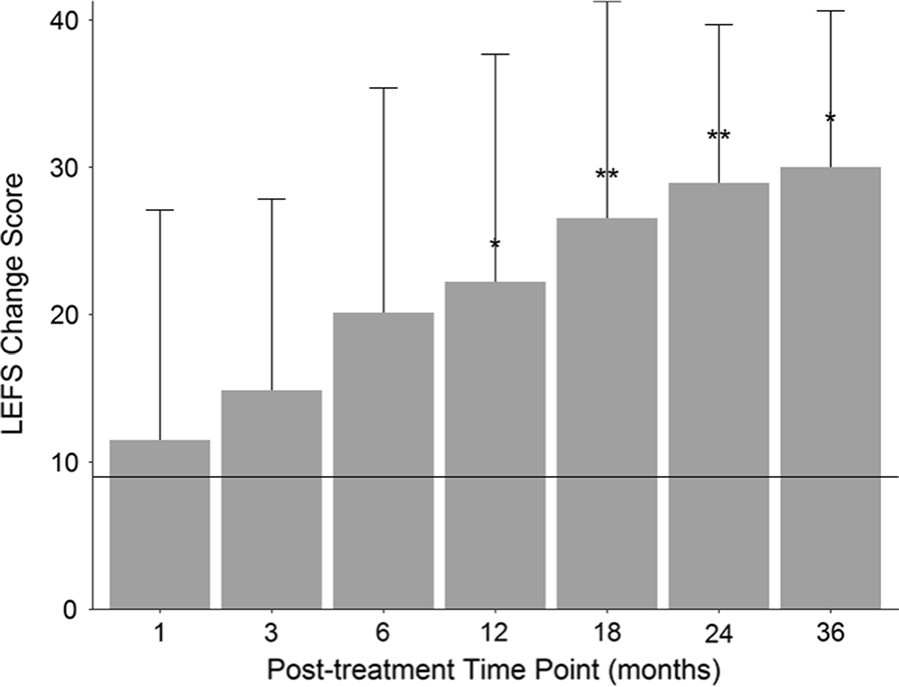
Mean LEFS change score at every post-treatment time-point. Scores were significantly higher than baseline at all time-points. MCID = 9 which is represented by horizontal line. *p < 0.05 compared to 1-month scores; **p < 0.05 compared to 1- and 3-month scores. Number of patients reporting at each time point: 1 month (N = 14); 3 month (N = 19); 6 month (N = 19); 12 month (N = 19); 18 month (N = 16); 24 month (N = 17); 36 month (N = 8)

Average baseline LEFS score was 51 (SD = 13). The last reported post-treatment LEFS average score was 73 (SD = 9) at an average of 23 months (SD = 10). The linear mixed-effects model showed scored differed across time (p < 0.0001), and post hoc Tukey showed mean LEFS scores were significantly higher than baseline at all time points (1, 3, 6, 12, 18, 24, and 36 months) post-treatment (p < 0.01). Further, scores at 12, 18, 24, and 36 months were all significantly higher than scores at 1-month post-treatment (p < 0.05) and scores at 18 and 24 months were significantly higher than scores at 3-months (p < 0.05). The minimal clinically important difference (MCID) for LEFS is 9, which was achieved at the last reported time point by 19 of the 23 patients with baseline LEFS information (83%) [21]. See Fig. 4 for a plot of the average LEFS change score at each time point. LEFS change scores did not significantly correlate with differences in ImageJ mean gray values (r = 0.37, p > 0.05).

### IKDC

Average baseline IKDC score was 53 (SD = 16). The last reported post-treatment IKDC average score was 86 (SD = 13) at an average of 23 months (SD = 10). Scores increased significantly across time points (p < 0.0001), with post hoc Tukey showing mean scores for IKDC were significantly higher at all post-treatment time points compared to baseline (p < 0.005), while 6, 12, 18, 24, and 36 month scores were all significantly higher than 1 month scores (p < 0.01), and 18, 24, and 36 month scores were significantly higher than 3-month scores (p < 0.05). The MCID for IKDC is 6.3 at a minimum of 6 months post-treatment, which 18 of 19 patients with reported scores achieved (95%); and the MCID is 16.7 at a minimum of 12 months post-treatment, which 14 of 14 patients with reported scores attained (100%). No significant correlations between IKDC change scores and MRI mean gray value differences were found (r = 0.12, p > 0.05). Table 2 displays the means for each outcome at all time points.

**Table 2:** Mean outcome scores at each time point, with number of patient reporting

	Baseline	1-month	3-month	6-month	12-month	18-month	24-month	36-month
Modified SANE		25.0 (14)	65.3^+^ (19)	75.5^+^ (19)	66.7^+^ (21)	78.8^+^ (16)	82.6^+^ (17)	88.8^+^ (8)
NPS	2.5 (25)	1.9 (15)	1.8 (20)	1.0* (19)	1.4 (19)	1.1* (16)	0.8* (18)	1.0 (8)
LEFS	51.1 (23)	61.4* (14)	65.7* (19)	72.0* (19)	72.2*^,+^ (19)	74.1*^,+^ (16)	75.9*^,+^ (17)	72.6*^,+^ (8)
IKDC	53.4 (20)	67.6* (14)	72.9* (18)	82.4*^,+^ (18)	80.1*^,+^ (19)	83.7*^,+^ (16)	87.0*^,+^ (18)	87.9*^,+^ (8)

Significant differences from baseline (*p < 0.05) and 1-month (^+^p<0.05)

### ACL injury grade

Anterior cruciate ligament injuries were broken into three tear grade groups, with 6 (21%) of patients with a grade 1 tear, 13 (45%) classified as grade 2, and 10 (34%) with grade 3 tears. Baseline scores for NPS, LEFS and IKDC were not significantly different between the three groups (p > 0.05). Mean last reported SANE ratings were higher in patients with grade 1 tears (93%) than patients with a grade 2 (74%) or 3 (69%), though not significantly (p > 0.05) Further, mean LEFS change score from the last reported time point to baseline was higher for the tear grade 1 group (29) compared to tear grade 2 (22) and grade 3 (16), though not significantly (p > 0.05).

### MRI ImageJ

**Fig. 5:**
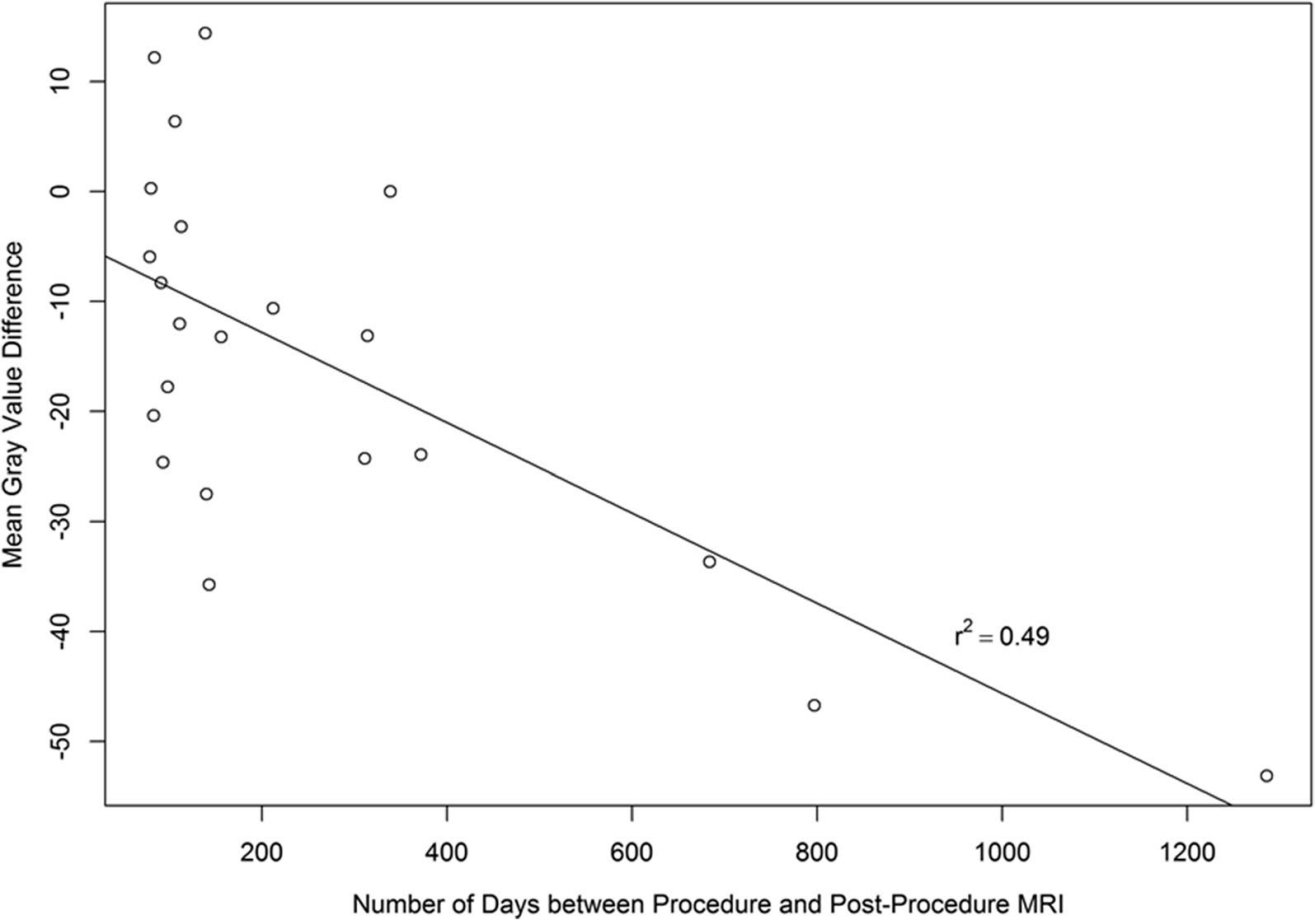
Mean Gray Value Difference vs. post-treatment MRI time-point

Post-treatment images in the current analysis were collected between 2.6 and 42.3 months after the procedure, with a mean of 8.7 months. The mean difference in A–P femoral head length between pre- and post-treatment MRIs was 0.47 mm (SD = 0.7) indicating the MRI slice matching was accurate and consistent. Seventy-seven percent of patients showed lower mean gray values post-treatment compared to before treatment. Gray value measurements of mean gray value, mode gray value, median gray value, and raw integrated density were all significantly lower (p < 0.01) in post-treatment MRIs compared to pre-treatment (Table 3). These lower signals suggest a more intact or dense ligament for post-treatment ACLs. Differences in mean gray value from pre- to post-treatment were plotted against the time point of post-treatment MRI, and a linear regression analysis was performed (r^2^ = 0.49, p < 0.01), shown in Fig. 5. The ANOVA analysis between pre-treatment, post-treatment and uninjured ACL mean gray value measurements showed a significant difference between groups (p < 0.001). Post-hoc Tukey showed that pre-treatment values were significantly higher than post-treatment (p < 0.05), as well as uninjured (p < 0.001) values. Further, post-treatment mean gray values were not significantly different than uninjured ACLs (p > 0.05). Pre-treatment mean gray values did not differ significantly between patients of different tear grades (p > 0.05).

**Table 3:** MRI ImageJ values

Variable	Mean	SD	Median	Lower quartile	Upper quartile	Minimum	Maximum	*p* value
Mean gray value	− 15.5	17.6	− 13.2	− 24.6	− 3.9	− 53.1	14.4	0.0015
Mode gray value	− 17.6	23.8	− 15.0	− 33.5	− 5.25	− 55.0	35.0	0.0025
Median gray value	− 15.6	19.0	− 13.5	− 26.5	− 0.5	− 54.0	19.0	0.0018
Skewness	0.16	1.0	0.2	− 0.4	0.8	− 2.4	2.3	0.3720
Raw integrated density	− 170,000	179,000	− 135,000	− 273,000	− 44,700	− 5,140,000	171,000	0.0003

p-values note significant differences in values from pre- to post-treatment. A negative value indicates a darker gray value which suggests more ligament healing

**Fig. 6:**
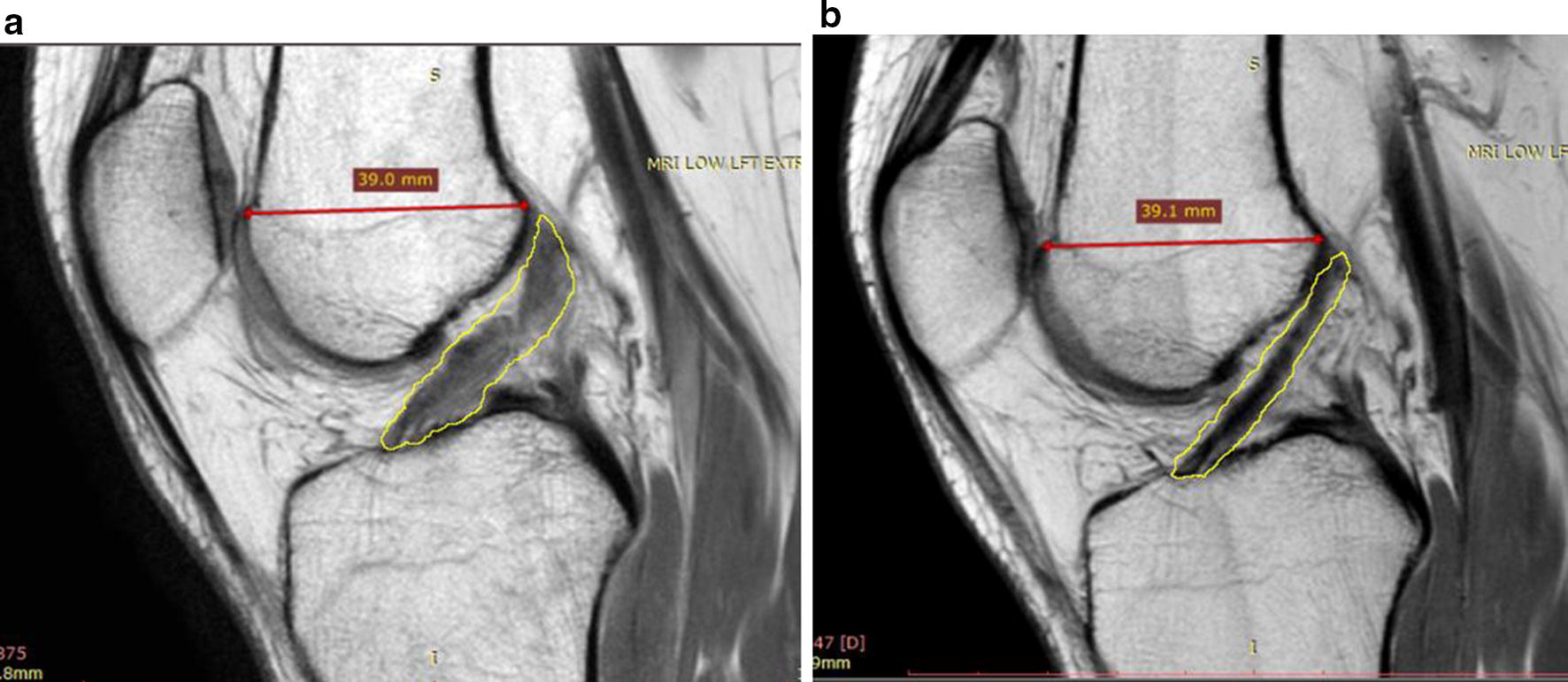
MRIs with ACL outlined using ImageJ software showing progression of ACL healing after BMC treatment. **a** Pre-treatment MRI showing injured ACL. **b** MRI at 22 months post-treatment displaying characteristics typical of an uninjured ACL (darker, more dense) indicating healing

**Fig. 7:**
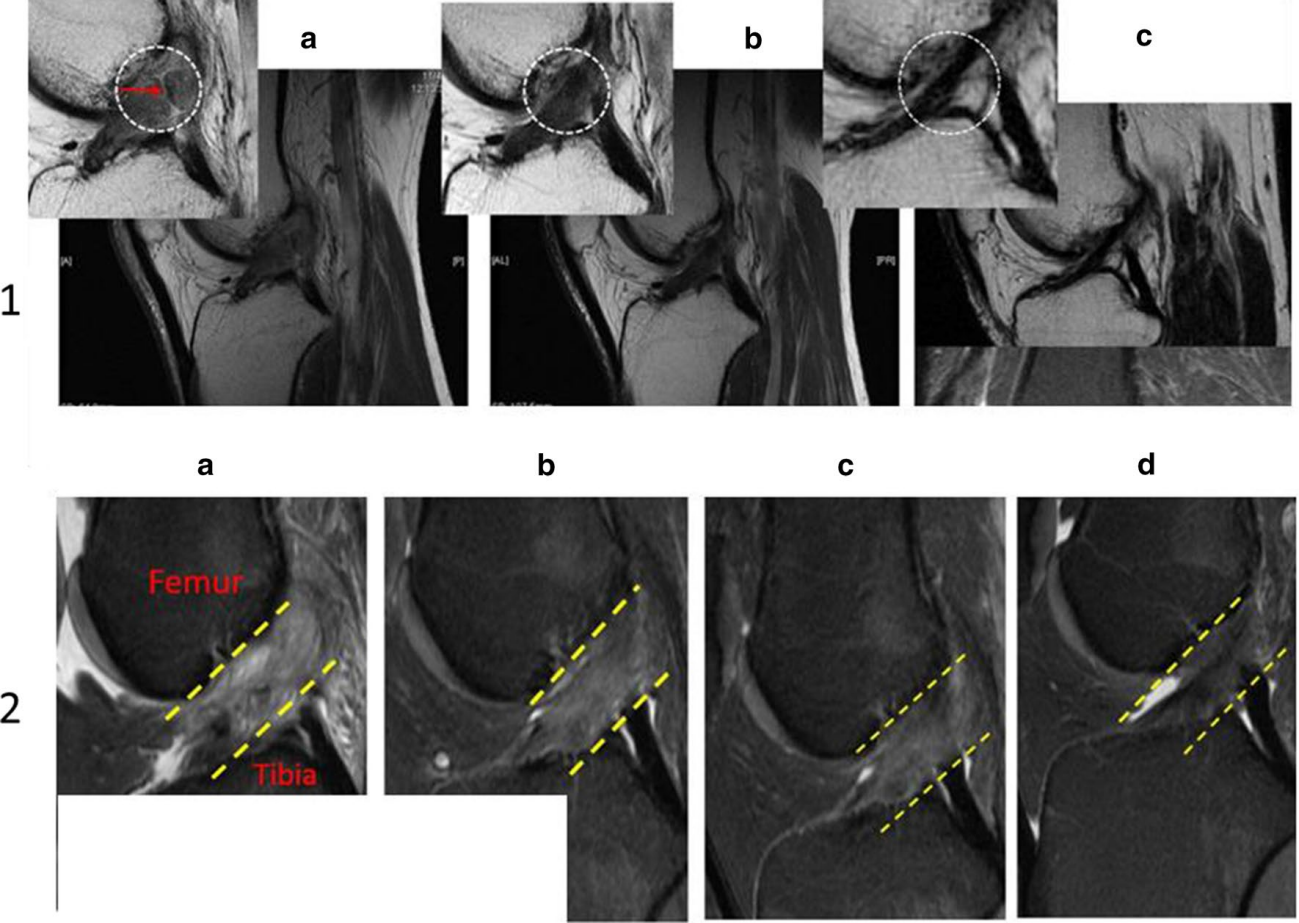
Progressions of ACL healing.** 1** (a) MRI images of ACL for patient before receiving treatment. (b) ACL at 5 months post-treatment (medium–low signal intensity). (c) ACL at 9 months post-treatment (low signal intensity).** 2** (a) MRI image of ACL pre-treatment. (b) ACL at 3 months post-treatment (medium signal intensity). (c) ACL at 6 months after stem cell procedure (medium–low signal intensity). (d) ACL at 11 months post-treatment (low signal intensity)

Figure 6 shows the pre- and post-treatment MRI images for a patient included in this study. The changes to the ACL are typical of the results observed on post-treatment imaging analysis. Figure 7 provides examples of a time series of post-treatment MRIs from two patients.

Gastrocnemius origin mean gray values did not differ between the 12 uninjured ACLs, 23 pre-treatment ACLs, and 23 post-treatment ACLs (p > 0.05). After normalization to the gastrocnemius origin, the ANOVA between the three groups showed significant differences were present (p < 0.01). Post-hoc Tukey showed pre-treatment ACL mean gray values still had a significantly higher signal than post-treatment ACLs (p < 0.05) and uninjured ACLs (p < 0.01). Further, no mean gray value differences between post-treatment ACLs and uninjured ACLs were detected (p > 0.05). After normalization, 77% of ACL images displayed lower mean gray values after treatment. Pre-treatment mean gray value differences did not differ significantly between tear grade groups (p > 0.05) after the normalization method was applied.

### Complications and adverse events

Two adverse events were reported. The first was swelling post-procedure and the second was a vasovagal episode, both of which resolved on their own. Five patients received ACL reconstruction surgeries (two were grade 1, two were grade 2, and one was grade 3). One was due to a re-tear and the others were treatment failures.

## Discussion

MRI findings consistent with ACL tear are well known and it is generally accepted that a normal ACL ligament has a low to medium signal intensity (i.e. darker appearance) with continuous fibers. Signs of an injured or torn ligament include high signal intensity (i.e. lighter appearance), a larger ligament cross-section, and fiber discontinuity [25]. In our study, the mean, median and mode gray values, as well as the raw integrated density, were significantly lower for post-treatment MRIs compared to pre-treatment MRIs. Over 75% of patients showed lower mean gray values after receiving treatment compared to baseline, which was validated by our normalization technique. This is consistent with imaging evidence of ACL healing. Skewness describes how the pixel density histogram is distributed relative to the mean, with a positive skew meaning the mass of distribution is concentrated on the left, indicating a darker image (see Fig. 2 histogram). Skewness was more positive, although not significantly, between pre- and post-treatment ACLs which could mean that although post-treatment ACLs were darker, some high signal is still present.

ImageJ data for the normal ACLs differed significantly from pre-treatment ACLs, both before and after normalization to the gastrocnemius origin, as hypothesized. Mean gray values between uninjured ACLs and post-treatment ACLs were not significantly different before nor after normalizing the data, indicating post-treatment ACLs were more in-tact and similar to normal ACLs. Figure 6 provides an example of a patients’ pre- and post-treatment imaging, depicting a darker and more dense ACL after BMC and platelet products treatment.

We also observed that MRI appearance of the treated ACLs continue to change over a number of months. Figure 7 shows a time series of post-treatment MRIs from two unique patients. These show the progression in healing from baseline up to 11 months post-treatment. The ACL MRI signal over time gets lower and denser at each time point, which is consistent with healing. This implies that MRI data can be considered a continuum where post-treatment MRI gray scale measurements may improve as time from treatment increases. For example, the average post-treatment MRI time point is 5.3 months (median = 3.7) with the exclusion of three outliers (23, 27, and 43 months). This may have contributed to an overall underestimation of healing for the patients in this study.

Improvements in modified SANE, LEFS and IKDC at later time points compared to scores at 1 and 3 months indicates continued healing over time. This is consistent with ligament and tendon healing clinical studies that show better clinical outcome after 3–6 months [26, 27]. Since this is a registry-based study that enrolled patients as they sought treatment over a period of several years, patients are at various post-treatment time points at the time of this publication. Hence, the number of patients reporting at the 36-month time point is the lowest (N = 8).

Patient reported measures reveal that patients improved in pain and function. The NPS analysis showed patients in our sample presented with an average of 2.5 out of 10 at baseline. Lower average pain scores were seen at all post-treatment time points compared to baseline, although the difference was not always statistically significant since starting pain was relatively low, leaving little room for improvement on this scale. A change of at least 9 points on the functional scale of LEFS designates a minimally clinically significant change, which 83% of the patients achieved at their last reported time point [21]. Scores of LEFS trended towards a gradual post-treatment improvement, with scores increasing 2–3 times the MCID for 6–36 months post-treatment. Scores for IKDC were found to increase gradually throughout the post-treatment time points overall. The MCID is presented and validated to be 6.3 at 6 months and 16.7 at 12 months post-treatment [28], which 95% and 100% of our patients met, respectively. Other studies have designated MCID as 11.5 [29, 30], which 90% of our patients attained. Our IKDC results are in line with other non-surgical interventions for ACL tears, which report increases in IKDC scores ranging from 10 to 18 points [31, 32]. Overall, the treated patients reported significantly better functional outcomes post-treatment relative to baseline.

Stratifying the data based on mean gray values and functional outcomes did not identify a correlation at the post-treatment time points (i.e. those with darker mean gray values did not have better functional outcome measures on the IKDC or LEFS). This may reflect the continued long-term healing that occurs even in the setting of lower mean gray values on MRI. Biercevicz et al. recently showed that at 5-year follow-up after ACL reconstruction, improved graft signal intensity and volume on MRI significantly correlated with patient reported and functional outcomes, which was not seen at the 3-year follow-up time point [33].

Of the 29 patients, eight returned for additional regenerative therapy treatments with autologous injections. A total of four repeat BMC with platelet product procedures were performed 5, 10, 12 and 18 months after the first, and a total of 8 platelet-rich-plasma (PRP) treatments were administered at 1 month, 4 months (3), 6 months, 9 months, 12 months, and 24 months. Additional analyses showed that those who did not receive additional injections reported significantly higher modified SANE scores (p < 0.01), had a greater drop in NPS scores (p < 0.01), and significantly better improvement in IKDC scores (p < 0.05) compared to those who did receive additional injections, suggesting that those who did not respond to treatment by improvement in pain and function were the patients who sought additional therapy.

Twenty-three patients had grade 2 and 3 tears, which are typically ACL reconstruction surgery candidates, of which 5 patients (22%) opted for surgery (17% of total sample population) occurring at 6 months (N = 1), 12 months (N = 2) and 24 months (N = 2). Only one patient experienced a re-tear during follow-up, which occurred after the patient returned to high-level sporting activities. In comparison, re-tears after ACL reconstruction have been found to occur in up to 10.4–29% of patients [34] and athletes who return to sport are 6 times more likely to re-injure themselves [34, 35]. In surgical studies, up to 29% of patients are shown to re-tear the ACL within 24 months [35]. The relatively low rate of patients opting for ACL reconstruction surgery and the low re-tear rate after receiving BMC and platelet products treatment are encouraging.

There are limitations and associated caveats with this case series. Limitations include the lack of randomization and control, placebo effect, missing data, the inclusion of the pre-injection, the use of PRP in conjunction with our BMC treatment, the absence of a standardized post-treatment rehabilitation program, and that post-procedure MRIs may not be accurately measuring tissue healing. This study did not include a randomization process with a control condition, which helps eliminate bias and quantify how much improvement may have been attributed to natural healing processes versus the BMC treatment, particularly in the patients with acute tears. Balancing this concern is the fact that grade 2 or 3 ACL injuries do not typically heal on their own [36]. Thus, our results were more likely to be associated with some aspect of the treatment [37–39]. We cannot rule out placebo effect for improvements in self-reported outcomes after receiving treatment, however, the findings observed on the post-treatment MRIs that are consistent with healing may indicate that the reported effects are actual. Missing data is an inherent issue with registry-based studies, which was addressed on the front end with repeated attempts for follow-up at each post-treatment time point, and on the back end by using statistical analyses that consider this. The inclusion of the pre-injection protocol may be considered a limitation, nevertheless, previous research has suggested that dextrose injections alone may help improve ACL laxity symptoms [41], increase vascular proliferation and thicken collagen [42]. Therefore, it cannot be ruled out that improvement may have been due to the pre-injection protocol, however, there is no research to support that dextrose injections can alter the appearance of an ACL on MRI. Additionally, eight subjects did not receive the pre-injection, and their outcomes did not differ significantly for any outcome metric. It is also possible that the PRP or PL included in the BMC therapy may account for the ACL changes observed on MRI. PRP injections have been shown to stimulate MSC proliferation and ACL cellular growth enhancement in vivo [40]. While possible, it has been previously demonstrated in a rabbit model that although PRP in combination with BMC does improve ACL integrity, PRP alone does not [41]. PRP contains growth factors that contribute to enhancing cellular proliferation and healing, but clinical trials using PRP to augment ligament and tendon repair have yielded mixed results [42]. Previous research does not show PRP to be effective as a primary treatment for ACL injuries [43, 44]. Patients were encouraged to participate in a post-procedural rehabilitation program and were provided a general activity protocol and a prescription for physical therapy. However, individual participation in therapy was not tracked nor accounted for in the results analysis. It should be noted that the changes noted on MRI could potentially represent scar tissue formation rather than healing in the ACL [45].

## Conclusion

The results of our case series suggest that a minimally invasive, percutaneous injection of bone marrow concentrate and platelet products into the ACL under fluoroscopic guidance may be a viable alternative to surgical ACL reconstruction for the treatment of grade 1, 2 and non-retracted grade 3 tears of the ACL. A larger randomized controlled trial is necessary to confirm these findings and examine whether the results from this study are attributable to the described treatment.

## Abbreviations

ACL: anterior cruciate ligament
ANOVA: analysis of variance
A–P: anterior–posterior
BMC: bone marrow concentrate
IKDC: International Knee Documentation Committee
IRB: Institutional Review Board
LEFS: Lower Extremity Functional Scale
MCID: minimal clinically important difference
MSC: mesenchymal stem cells
MRI: magnetic resonance imaging
NPS: numeric pain scale
OA: osteoarthritis
PL: platelet lysate
PRP: platelet-rich plasma
ROI: region of interest
SANE: Single Assessment Numeric Evaluation
SD: standard deviation
TNCC: total nucleated cell count

## References

[1] Siegel L, Vandenakker-Albanese C, Siegel D (2012). Anterior cruciate ligament injuries: anatomy, physiology, biomechanics, and management. Clin J Sport Med.

[2] Brophy RH, Wright RW, Matava MJ (2009). Cost analysis of converting from single-bundle to double-bundle anterior cruciate ligament reconstruction. Am J Sports Med.

[3] Herzog MM, Marshall SW, Lund JL, Pate V, Spang JT (2017). Cost of outpatient arthroscopic anterior cruciate ligament reconstruction among commercially insured patients in the United States, 2005–2013. Orthop J Sports Med..

[4] Spindler KP, Warren TA, Callison JC, Secic M, Fleisch SB, Wright RW (2005). Clinical outcome at a minimum of five years after reconstruction of the anterior cruciate ligament. J Bone Joint Surg Am.

[5] Lohmander LS, Englund PM, Dahl LL, Roos EM (2007). The long-term consequence of anterior cruciate ligament and meniscus injuries: osteoarthritis. Am J Sports Med.

[6] Pujol N, Colombet P, Cucurulo T, Graveleau N, Hulet C, Panisset JC (2012). Natural history of partial anterior cruciate ligament tears: a systematic literature review. Orthop Traumatol Surg Res..

[7] Andersson C, Odensten M, Good L, Gillquist J (1989). Surgical or non-surgical treatment of acute rupture of the anterior cruciate ligament. A randomized study with long-term follow-up. J Bone Joint Surg Am.

[8] Simon D, Mascarenhas R, Saltzman BM, Rollins M, Bach BR, MacDonald P (2015). The relationship between anterior cruciate ligament injury and osteoarthritis of the knee. Adv Orthop.

[9] Li RT, Lorenz S, Xu Y, Harner CD, Fu FH, Irrgang JJ (2011). Predictors of radiographic knee osteoarthritis after anterior cruciate ligament reconstruction. Am J Sports Med.

[10] Nomura Y, Kuramochi R, Fukubayashi T (2015). Evaluation of hamstring muscle strength and morphology after anterior cruciate ligament reconstruction. Scand J Med Sci Sports.

[11] Bonfim TR, Jansen Paccola CA, Barela JA (2003). Proprioceptive and behavior impairments in individuals with anterior cruciate ligament reconstructed knees. Arch Phys Med Rehabil.

[12] Barrett DS (1991). Proprioception and function after anterior cruciate reconstruction. J Bone Joint Surg Br.

[13] Centeno CJ, Pitts J, Al-Sayegh H, Freeman MD (2015). Anterior cruciate ligament tears treated with percutaneous injection of autologous bone marrow nucleated cells: a case series. J Pain Res..

[14] Hao ZC, Wang SZ, Zhang XJ, Lu J (2016). Stem cell therapy: a promising biological strategy for tendon-bone healing after anterior cruciate ligament reconstruction. Cell Prolif.

[15] Fu W, Li Q, Tang X, Chen G, Zhang C, Li J (2016). Mesenchymal stem cells reside in anterior cruciate ligament remnants in situ. Int Orthop.

[16] Hong SH, Choi JY, Lee GK, Choi JA, Chung HW, Kang HS (2003). Grading of anterior cruciate ligament injury. Diagnostic efficacy of oblique coronal magnetic resonance imaging of the knee. J Comput Assist Tomogr.

[17] Schallmoser K, Strunk D (2009). Preparation of pooled human platelet lysate (pHPL) as an efficient supplement for animal serum-free human stem cell cultures. J Vis Exp..

[18] Huang YL, Qiu RF, Mai WY, Kuang J, Cai XY, Dong YG (2012). Effects of insulin-like growth factor-1 on the properties of mesenchymal stem cells in vitro. J Zhejiang Univ Sci B..

[19] Zhang X, Zhang Y, Wang Z, Li Q, Li B (2014). The effect of non-growth factors on chondrogenic differentiation of mesenchymal stem cells. Cell Tissue Bank..

[20] Steilen D, Hauser R, Woldin B, Sawyer S (2014). Chronic neck pain: making the connection between capsular ligament laxity and cervical instability. Open Orthop J..

[21] Binkley JM, Stratford PW, Lott SA, Riddle DL (1999). The Lower Extremity Functional Scale (LEFS): scale development, measurement properties, and clinical application. North American Orthopaedic Rehabilitation Research Network. Phys Ther.

[22] Hefti F, Muller W, Jakob RP, Staubli HU (1993). Evaluation of knee ligament injuries with the IKDC form. Knee Surg Sports Traumatol Arthrosc.

[23] Shelbourne KD, Barnes AF, Gray T (2012). Correlation of a single assessment numeric evaluation (SANE) rating with modified Cincinnati knee rating system and IKDC subjective total scores for patients after ACL reconstruction or knee arthroscopy. Am J Sports Med.

[24] Centeno CJ, Elliott J, Elkins WL, Freeman M (2005). Fluoroscopically guided cervical prolotherapy for instability with blinded pre and post radiographic reading. Pain Physician..

[25] Ng WH, Griffith JF, Hung EH, Paunipagar B, Law BK, Yung PS (2011). Imaging of the anterior cruciate ligament. World J Orthop..

[26] Creaney L, Wallace A, Curtis M, Connell D (2011). Growth factor-based therapies provide additional benefit beyond physical therapy in resistant elbow tendinopathy: a prospective, single-blind, randomised trial of autologous blood injections versus platelet-rich plasma injections. Br J Sports Med.

[27] Sanchez M, Anitua E, Azofra J, Andia I, Padilla S, Mujika I (2007). Comparison of surgically repaired Achilles tendon tears using platelet-rich fibrin matrices. Am J Sports Med.

[28] Greco NJ, Anderson AF, Mann BJ, Cole BJ, Farr J, Nissen CW, Irrgang JJ (2010). Responsiveness of the International Knee Documentation Committee Subjective Knee Form in comparison to the Western Ontario and McMaster Universities Osteoarthritis Index, modified Cincinnati Knee Rating System, and Short Form 36 in patients with focal articular cartilage defects. Am J Sports Med.

[29] Irrgang JJ, Anderson AF, Boland AL, Harner CD, Neyret P, Richmond JC, Shelbourne KD (2006). Responsiveness of the international knee documentation committee subjective knee form. Am J Sports Med.

[30] Wright RW (2009). Knee injury outcomes measures. J Am Acad Orthop Surg.

[31] Ha CW, Cho JJ, Elmallah RK, Cherian JJ, Kim TW, Lee MC, Mont MA (2015). A multicenter, single-blind, phase IIa clinical trial to evaluate the efficacy and safety of a cell-mediated gene therapy in degenerative knee arthritis patients. Hum Gene Ther Clin Dev..

[32] Tsai LC, Lee SJ, Yang AJ, Ren Y, Press JM, Zhang LQ (2015). Effects of off-axis elliptical training on reducing pain and improving knee function in individuals with patellofemoral pain. Clin J Sport Med.

[33] Biercevicz AM, Akelman MR, Fadale PD, Hulstyn MJ, Shalvoy RM, Badger GJ (2015). MRI volume and signal intensity of ACL graft predict clinical, functional, and patient-oriented outcome measures after ACL reconstruction. Am J Sports Med.

[34] Wright RW, Magnussen RA, Dunn WR, Spindler KP (2011). Ipsilateral graft and contralateral ACL rupture at five years or more following ACL reconstruction: a systematic review. J Bone Joint Surg Am.

[35] Paterno MV, Rauh MJ, Schmitt LC, Ford KR, Hewett TE (2014). Incidence of second ACL injuries 2 years after primary ACL reconstruction and return to sport. Am J Sports Med.

[36] Woo SL, Chan SS, Yamaji T (1997). Biomechanics of knee ligament healing, repair and reconstruction. J Biomech.

[37] Bray RC, Leonard CA, Salo PT (2003). Correlation of healing capacity with vascular response in the anterior cruciate and medial collateral ligaments of the rabbit. J Orthop Res.

[38] Rizzello G, Longo UG, Petrillo S, Lamberti A, Khan WS, Maffulli N, Denaro V (2012). Growth factors and stem cells for the management of anterior cruciate ligament tears. Open Orthop J..

[39] Woo SL, Niyibizi C, Matyas J, Kavalkovich K, Weaver-Green C, Fox RJ (1997). Medial collateral knee ligament healing. Combined medial collateral and anterior cruciate ligament injuries studied in rabbits. Acta Orthop Scand.

[40] Dhillon MS, Karna SK, Dhatt SS, Behera P, Bhatia A (2015). Can platelet rich plasma stimulate human ACL growth in culture? A preliminary experience. Muscles Ligaments Tendons J..

[41] Teng C, Zhou C, Xu D, Bi F (2016). Combination of platelet-rich plasma and bone marrow mesenchymal stem cells enhances tendon-bone healing in a rabbit model of anterior cruciate ligament reconstruction. J Orthop Surg Res..

[42] Yuan T, Zhang CQ, Wang JH (2013). Augmenting tendon and ligament repair with platelet-rich plasma (PRP). Muscles Ligaments Tendons J..

[43] Grambart ST (2015). Sports medicine and platelet-rich plasma: nonsurgical therapy. Clin Podiatr Med Surg.

[44] Moraes VY, Lenza M, Tamaoki MJ, Faloppa F, Belloti JC (2014). Platelet-rich therapies for musculoskeletal soft tissue injuries. Cochrane Database Syst Rev..

[45] Higueras GV, Torregrosa AA, Marti-Bonmati L, Casillas C, Sanfeliu M (1999). Synovialisation of the torn anterior cruciate ligament of the knee: comparison between magnetic resonance and arthroscopy. Eur Radiol.

